# Development of a Laboratory Model of a Phototroph-Heterotroph Mixed-Species Biofilm at the Stone/Air Interface

**DOI:** 10.3389/fmicb.2015.01251

**Published:** 2015-11-17

**Authors:** Federica Villa, Betsey Pitts, Ellen Lauchnor, Francesca Cappitelli, Philip S. Stewart

**Affiliations:** ^1^Center for Biofilm Engineering, Montana State University, BozemanMT, USA; ^2^Dipartimento di Scienze per gli Alimenti, la Nutrizione e l’Ambiente, Università degli Studi di MilanoMilano, Italy

**Keywords:** subaerial biofilms, stone monuments, Lab-scale system, phototroph-heterotroph interactions, dual-species subaerial biofilm

## Abstract

Recent scientific investigations have shed light on the ecological importance and physiological complexity of subaerial biofilms (SABs) inhabiting lithic surfaces. In the field of sustainable cultural heritage (CH) preservation, mechanistic approaches aimed at investigation of the spatiotemporal patterns of interactions between the biofilm, the stone, and the atmosphere are of outstanding importance. However, these interactions have proven difficult to explore with field experiments due to the inaccessibility of samples, the complexity of the ecosystem under investigation and the temporal resolution of the experiments. To overcome these limitations, we aimed at developing a unifying methodology to reproduce a fast-growing, phototroph-heterotroph mixed species biofilm at the stone/air interface. Our experiments underscore the ability of the dual-species SAB model to capture functional traits characteristic of biofilms inhabiting lithic substrate such as: (i) microcolonies of aggregated bacteria; (ii) network like structure following surface topography; (iii) cooperation between phototrophs and heterotrophs and cross feeding processes; (iv) ability to change the chemical parameters that characterize the microhabitats; (v) survival under desiccation and (vi) biocide tolerance. With its advantages in control, replication, range of different experimental scenarios and matches with the real ecosystem, the developed model system is a powerful tool to advance our mechanistic understanding of the stone-biofilm-atmosphere interplay in different environments.

## Introduction

Microbiologists working in the field of cultural heritage (CH) are faced with the challenge of understanding the physiology and the activity of biofilms inhabiting outdoor stone heritage (subaerial biofilms, SABs), and their complex interactions with the mineral substrate and the atmosphere at different spatial and temporal scales (Gorbushina, 2007; Pinna, 2014).

The approaches used to explore such complexity rely mainly on field investigations (Polo et al., 2012; Villa et al., 2015). Although field experiments are undoubtedly instrumental in understanding the relationship between SABs and ecosystem properties, their experimental design and execution are hampered by a number of challenges. These challenges include limited and extremely small samples, complexity of sample structure, the importance of maintaining spatial integrity and inaccessibility to repeated sampling (Owens et al., 2014). Furthermore, the results of many field-based studies are limited by the temporal resolution of the experiments, as many processes that might be important in structuring SAB communities and activity, such as succession, coevolution, invasion, and climate change occur over a longer time scale than those of the average research grant, limiting the understanding of the phenomena under investigation.

In the light of the previous considerations, the simplicity of model systems provides a stark contrast to the complexity and inaccessibility of the environmental system, enabling researchers to test hypotheses about the physiology of SAB on stone, and to establish the plausibility of mechanisms governing the biogeochemical processes occurring in the field (Jessup et al., 2004).

Despite the significance of model systems in CH studies, the development and explicit use of microbial lab-scale systems has been relatively rare, and most of them are experimental model systems of phototrophic biofilms (Ortega-Calvo et al., 1991; Guillitte and Dreesen, 1995; Monte, 2003; Prieto and Silva, 2005; Miller et al., 2008; Sanmartín et al., 2011, 2015). In fact, within the list of the most typical stone colonizers, cyanobacteria have received particular attention as they adapt to extremes of environmental stress and they are able to readily colonize a wide variety of terrestrial habitats, including modern and ancient buildings, sometimes causing extensive esthetic, physical and chemical damages (Crispim and Gaylarde, 2005; Cappitelli et al., 2012). In most of these works, the individual members of the phototrophic community were studied separately, precluding the investigation of species interactions. Miller et al. (2008, 2009) cultivated a natural green biofilm from an enriched microbial consortium residing on a limestone monument. The biofilm was grown over a 3-month period under laboratory conditions in a custom chamber, which exposes stone samples to intermittently sprinkling water. These biofilm studies showed complex microbial communities, simulating the existence of competition and/or synergy between colonizing microorganisms (Miller et al., 2008, 2009).

Despite the success in reproducing complex phototrophic SABs, diagnostic and prognostic tools for biofilm studies on different materials and under different environmental conditions require microorganisms with available genetic and physiological information (Noack-Schönmann et al., 2014), features that are rarely encountered in complex microbial consortia isolated from the field. Gorbushina and Broughton (2009) proposed a model biofilm comprising the cyanobacterium *Nostoc punctiforme* strain ATCC 29133 (PCC 73102) as phototroph, and the well-studied marble-derived isolated microcolonial fungus A95 *Knufia petricola* (syn. *Sarcinomyces petricola*; Nai et al., 2013) as heterotrophic component of the dual-species consortium. Mixed cyanobacterial/fungal biofilms were grown on membrane filters placed on top of agarized media without carbon and nitrogen sources. In this case, the biofilm was grown at the agar/air interface following the colony biofilm method, which simulates a no-shear environment (Anderl et al., 2000). Seiffert et al. (2014) used the same well-characterized consortium to test the mineral weathering potential of mono and dual-species biofilms grown at the solid/liquid interface. Minerals with different grain sizes and mineralogy were incubated with and without biofilm in batch and in flow through experiments over a 5-month period. The biofilm was exposed to an organic-carbon-rich environment, being fed with a nutrient medium.

Although many authoritative scientific works have successfully embraced microbial model systems as tools to address questions related to the biological colonization of lithic substrate, none of them offer the opportunity in one single system to reproduce a fast-growing, phototroph-heterotroph mixed species biofilm at the stone/air interface.

To overcome these limitations, we aimed at developing a unifying methodology to obtain a laboratory model of SAB biofilm able to mirror the main features of biofilms inhabiting lithic substrate, while keeping simplicity and high degrees of experimental control. More specifically, we established a dual-species biofilm model system that incorporates the following characteristics: (i) stone/air interface, (ii) phototroph-heterotroph interactions, (iii) oligotrophic environment, (iv) microorganisms well-characterized, amenable to genetic manipulation and with already developed *in silico* metabolic models, (v) discontinuous low-shear/laminar flow and high gas transfer environment and (vi) fast-growing biofilm.

These results showed the efficacy of the system in reproducing SABs, being able to capture features typical of biofilms on outdoor stone monuments such as: (i) microcolonies of aggregated bacteria; (ii) network-like structure following surface topography; (iii) cooperation between phototrophs and heterotrophs and cross feeding processes; (iv) ability to change the chemical parameters that characterize the microhabitats; (v) survival in harsh environment including desiccation stress and (vi) biocide tolerance.

To the best of our knowledge, this is the first time that a phototroph-heterotroph association at the stone/air interface has been successfully obtained at laboratory scale starting from two introduced, controlled species and not from an environmental microbial consortium. The present study has the potential to significantly advance our mechanistic understanding of the biofilm-stone-air interplay that has proven difficult to study in field experiments due to the inaccessibility of samples and the complexity of the ecosystem under investigation.

## Materials and Methods

### Laboratory Strains and Culturing Conditions

Axenic batch cultures of the photoautotrophic bacterium *Synechocystis* PCC 6803 (ATCC 27184) were routinely grown in BG11 medium (Sanmartín et al., 2011). The cultivation was carried out at room temperature in a 250-mL Erlenmeyer flasks on a standard orbital shaker, under a 14/10 day/night photoperiod and 40 μmol (photons) m^-2^ s^-1^ illumination over a period of 17 days. Axenic cultures of GFP-*Escherichia coli* K12 MG1655 were grown overnight in M9 medium (Harwood and Cutting, 1990) amended with 10 g l^-1^ glucose and supplemented with 100 mg l^-1^ ampicillin at 37°C.

### Subaerial Biofilms (SABs) Growth using the Drip Flow Biofilm Reactor

A modified Drip Flow Reactor (DFR, Biosurface Technology Corp., USA) with a glass lid was used in this study to reproduce SABs at the stone/air interface. To initiate biofilm growth, individual stationary-phase cultures of *Synechocystis* PCC 6803 and *E. coli* K12 were centrifuge at 3500 rpm at room temperature for 15 min, rinsed two times with BG11 and then resuspended in the same medium. The axenic cultures were adjusted to obtain a cell concentration of approximately 10^8^ cell ml^-1^. Next, a volume of each of the *Synechocystis* and *E. coli* cultures were mixed, and the mono-cultures diluted 1:2 with BG11 to obtain a final cell concentration of approximately 5 × 10^7^ cell ml^-1^ for each microorganisms in both mono and co-cultures. Twenty ml of the mixed planktonic culture was added to each channel, which also held a 0.5 cm thick limestone tile cut to the dimensions of a microscope slide. The reactor was placed in a flat, level position on the bench top and left for 24 h at room temperature under a 14/10 day/night photoperiod of 40 μmol(photons) m^-2^ s^-1^ illumination (the light cycle began with inoculation of the stone coupons). After 24 h of batch conditions, the reactor was set on a surface held at a 10° slant and attached to the medium reservoir. The medium (100%-strength BG11) was pumped through the system every 12 min at 1 ml min^-1^ for 3 min, creating a discontinuous flow rate. The reactor operated in discontinuous flow mode for 10 days at room temperature under a 14/10 day/night photoperiod and 40 μmol(photons) m^-2^ s^-1^ illumination. Every 48 h, biofilms were sampled for either plate-count enumeration or microscopy investigations. Biofilm growths were repeated multiple times (N: 5) using a separate inoculum mixture.

### Biomass Quantification by Plate Counting

Viable cell counts for each species within the established mono and dual-species SABs were determined using selective agar. Every 2 days, the stone coupons were aseptically removed from the reactor and placed into 50-ml Falcon tubes containing 10 ml phosphate buffered saline (PBS, 10 mM phosphate buffer, 0.3 M NaCl pH 7.4 at 25°C, Sigma–Aldrich, USA). Sessile cells were dislodged from the stone coupon by brushing off the surface with a sterile toothbrush. The dislodged biofilms were homogenized (IKA T25 Ultra Turrax) at 10000 rpm for 30 s followed by 30 s vortex mixing. The biofilm suspension was then serially diluted in PBS and drop-plated (Herigstad et al., 2001) on agarized BG11 (Noble agar, Fisher Scientific, USA) and TSA, to isolate and enumerate *Synechocystis* and *E. coli*, respectively. The plates were then counted, and the number of CFU per cm^2^ was calculated for each microorganism. The maximum specific growth rates (μm) of each bacterium in both mono and dual-species SABs were estimated from the CFU cm^-2^ data vs. time (days) as reported by Cattò et al. (2015). Experiments were performed in triplicate.

### *Escherichia coli* Growth on Cyanobacterial Extracellular Polymeric Substances (EPS)

Mature mono-species cyanobacterial SABs (*n* = 6 stone coupons combined in one sample) were collected and resuspended in 2 ml 2% ethylenediaminetetraacetic acid (EDTA, Sigma–Aldrich, USA). Biofilm cell suspensions were first gently sonicated and then shaken at 300 rpm for 3 h at 4°C. After incubation, the samples were centrifuged for 20 min, 5000 rpm at 4°C to separate the supernatants containing the EPS from the cell pellets. EPS was recovered from filtered supernatant (0.45 μm, Fisher Scientific, USA) after overnight precipitation with two volumes of chilled ethanol at -20°C, centrifugation at 13000 rpm for 30 min at 4°C, washing with 95% ethanol, drying under air, and re-suspension in 380 μl M9 mineral medium. This volume corresponded to the biofilm volume estimated as follows: (area of the stone coupons, 11856 mm^2^) × (average of the biofilm thickness, 0.032 mm). Total carbohydrate contents of EPS were measured by the phenol-sulfuric method, and the amount of EPS was calculated as the average ratio of the EPS quantity over the dry biomass as reported by Villa et al. (2012).

Planktonic growth of *E. coli* on EPS solutions was carried out in 96-well microtiter plates. Growth curves at room temperature were generated using the Synergy HT microplate reader (Biotek, USA). The growth was followed by measuring the absorbance at 600 nm (OD600) every 10 min for over 17 h in wells inoculated with 3 μl of an overnight *E. coli* culture (final concentration 10^7^ cells ml^-1^). Experiments were performed in triplicate.

### Biofilm Imaging by Confocal Laser Scanning Microscopy (CLSM) and Field Emission Scanning Electron Microscopy (FE-SEM)

The development and the structure of the dual-species SABs were monitored by CLSM and FE-SEM. For CLSM analyses, samples were stained with the lectin ConA-Texas Red conjugate (Invitrogen, USA) as reported by Villa et al. (2012). Confocal images were collected using a Leica TCS-SP5 confocal microscope (Leica Microsystems Heidelberg GmbH, Germany) and a 40× 0.7NA 3.3 mm WD water immersion objective. Fluorescence was excited and collected using the following laser lines and emission parameters: for GFP-tagged *E. coli* cells, ex 488 nm, em 500–550 nm ConA-Texas Red ex 561 nm, em 570–620 nm, and autofluorescence of *Synechocystis* ex with 633 nm, em 650–750 nm. In addition, the CLSM was used in reflectance mode with the 488 nm argon line for relief imaging of specimens. Captured images were analyzed with the software Imaris (Bitplane Scientific Software, Switzerland) for 3D reconstructions of SABs.

Field Emission Scanning Electron Microscopy analyses of SAB samples were carried out using a Zeiss SUPRA 55VP (Zeiss, Oberkochen, Germany) at an acceleration voltage of 1 kV using the Everhart-Thornley SE-detector and the inlens SE-detector in a 25:75 ratio.

A minimum of three biofilm samples was analyzed and representative images are presented.

### Biofilm Cryosectioning and Thickness Measurements

Subaerial biofilms cryosectioning and thickness measurements were conducted as reported by Villa et al. (2012).

Briefly, SABs on limestone coupons were carefully covered with a layer of OCT (Tissue-Tek Optimum Cutting Temperature, VWR Scientific, USA) and placed on dry ice until completely frozen. The frozen samples were sectioned at -19°C using a Leica CM1850 cryostat (Leica Microsystems Heidelberg GmbH, Germany), and the 5-μm thick cryosections were mounted on Superfrost/Plus microscope slides (Fisher Scientific, USA).

Sections were observed using a Nikon Eclipse E800 microscope with a 20× dry. The sections were viewed in the epifluorescence mode with red (to visualize *Synechocystis* cells) and green (to visualize GFP-*E. coli* cells) filters. The software MetaMorph (Molecular Devices, Downingtown, PA) performed the image analysis and biofilm thickness measurements. More than five images per sample were taken for microscope analysis. For each picture, the biofilm thickness was measured at three different locations randomly selected along the profile. These measurements were used to calculate the average thickness and the associated standard deviation. The experiment was conducted in triplicate.

### Oxygen and pH Microsensors Analyses

Dual-species SABs were grown as previously described on agar-coated slice coupons to allow the use of microelectrodes that otherwise would be hampered by the presence of the stone substrate. The local concentrations of O_2_ and pH at different locations on the biofilm surface were measured over time to analyze the response of the biofilms to light and dark conditions.

The microelectrodes for oxygen (OX-10, Unisense, Denmark) and pH (pH-25, Unisense, Denmark) were connected to a millivolt meter (Unisense, Denmark) for voltage supply and signal acquisition. A micromanipulator with motor controller (MM-2, Unisense, Denmark) was used to position the microelectrodes in the SAB. Data acquisition was performed on a laptop computer connected to the multimeter, using SensorTrace Pro software (Unisense, Denmark).

Oxygen microsensor measurements were performed using Clark-type oxygen microelectrodes with tip diameters of 10 μm, described in detail elsewhere (Revsbech, 1989). A two-point calibration was performed for O_2_ sensors using medium saturated with dissolved O_2_ for a 100% O_2_ saturated value and medium sparged with pure N_2_(g) for at least 30 min for a zero value. Calibrations were repeatedly checked in the anoxic standard and in air-saturated diH_2_O throughout the experiments. Microsensor measurements were performed at room temperature under both dark and light conditions (PAR = 40 μmol photons m^-2^ s^-1^) in presence of the liquid medium BG11. Before the microsensor measurements, the SAB was incubated 20–25 min in the dark at room temperature.

For the pH measurements, a pH microelectrode was used with a tip diameter of 25 μm in combination with an open-ended Ag-AgCl reference microelectrode with a tip diameter of 25 μm (REF-25; Unisense, Denmark). The sensor was linearly calibrated from signal readings in pH standard buffers of 4.0, 7.0, and 10.0 at the experimental temperature. The pH measurements were conducted as described for the oxygen microsensor. The reference electrode was placed in the micromanipulator and lowered into the biofilm alongside the pH electrode, to ensure fluid contact between the pH and reference electrode during the measurements.

Microelectrode measurements were taken in three different spots of two different SABs. During time resolved measurements of O_2_ and pH change during light/dark shifts, measurements were recorded at a single location at an interval of 5 s.

### Desiccation Experiment and Live Cell Imaging of Biofilm Recovery

Mature mono- and dual-species SABs were left in the reactor channels. Influent tubings were attached to air supply systems composed of common aquarium air pumps, ultrafilter membranes and rubber tubing. Air entered at the top of each single reactor channel and exited at the bottom through the eﬄuent port, without being pressurized in the DFR. The idea was to expose the SABs grown on stone to constant breeze and extreme dry conditions over 1 h. After that time, the samples were removed from the reactor and inserted into an environmental control chamber (Pathology Devices LiveCell+ system, USA) to control both the temperature and the humidity. The chamber was mounted on the motorized stage of an inverted Leica TCS SP5 confocal microscope (Leica Microsystems Heidelberg GmbH, Germany). The CLSM control software was set to take a series of time-lapse xyzt scans at intervals of 5 min at different depths in the biofilm over a period of 90 min. The environmental chamber was set at 25°C, and the relative humidity (RH) was gradually increased from 28 to 90%.

Biofilms were scanned at 600 Hz using a 10× dry objective with a 488 and 633 nm laser excitation lines to visualize both green GFP-*E. coli* cells and the red autofluorescence of the phototrophic component of the mono- and dual-species SAB. Images were analyzed in MetaMorph software (Universal Imaging Corp., Downington, PA) in order to track the average fluorescence intensity of the entire image for each channel. Average intensity values were normalized by dividing the fluorescence intensity recorded at the different time points by the initial average fluorescence intensity values.

The emission spectra of cyanobacterial pigments were obtained using a wavelength λ-scan function of the CLSM as reported by Roldán et al. (2014). Region of interest (ROIs) representing single cells were used to obtain fluorescence spectra. The fluorescence spectra were analyzed by Pickfit deconvolution software (PeakFit, SPSS, Inc.) to resolve individual phycobiliproteins as reported by Wolf and Schübler (2005). Representative fluorescence spectra of dehydrated and rewetted cells within the biofilm are presented.

### Antimicrobial Effectiveness of a Biocide Solution against Planktonic and Biofilm Cells

A 12-day mature dual-species biofilm was challenged with the quaternary ammonium solution D/2 (D/2 Biological Solutions, Inc., USA), a biocide frequently used in restoration of architectural surfaces including monuments, sculpture and headstones.

The undiluted D/2 was dripped onto the stone coupons to wet the entire surface with the biocide solution. D/2 was allowed to remain on the surface for 15 min at room temperature as per manufacturer’s instructions. After the contact period, the coupons were rinsed with water and transferred to Falcon tubes for colony counting.

Planktonic cell experiments were performed with a mixed culture of *Synechocystis* and *E. coli* to simulate conditions in dual-species biofilm experiments. Stationary-phase cultures of the individual two species were centrifuged at 3500 rpm for 15 min at room temperature and then resuspended in BG11 at the final concentration of 10^8^ CFU ml^-1^ for each of the species. Antimicrobial agent solutions were added to the planktonic cultures as for the biofilm experiments. Planktonic cells were treated for 15 min at room temperature. The disinfection efficacy of each sample was evaluated by plate counting as described for biofilm experiments. Antimicrobial efficacy was expressed as log_10_ reduction in the microbial survival. The log_10_ reduction was calculated relative to the cell count in the control samples without biocides. The antimicrobial experiments were conducted in triplicate.

The susceptibility of the dual-species SAB to D/2 was also evaluated by time lapse CLSM. This technique permits the direct visualization of cell inactivation patterns in biofilm structure during biocide action. Fluorescence loss from GFP *E. coli* cells and autofluorescence loss from *Synechocystis* cells were used to monitor real-time loss in cell viability. The stone coupon hosting the dual-species SAB and exposed to undiluted D/2 was mounted on the motorized stage of an inverted Leica SP5. Biofilms were scanned at 600 Hz using a 10× dry objective as previously described in the “Desiccation experiment and live cell imaging of biofilm recovery” section. Biofilms were then scanned every 3 min at different depths over 45 min, and both red and green fluorescence loss within the structure was recorded. The overall effect of a treatment on SAB was assessed by tracking the average fluorescence intensity of the entire image for each channel. A control test was also performed in order to quantify the fluorescence lost by photobleaching.

### Statistical Analysis

Analysis of variance (ANOVA) via a software run in MATLAB environment (Version 7.0, The MathWorks Inc., USA) was applied to statistically evaluate any significant differences among the samples. Tukey’s honestly significant different test (HSD) was used for pairwise comparison to determine the significance of the data. Differences were considered significant for *p* < 0.05.

## Results And Discussion

### Mono- and Dual-species Biofilm Growth

The unicellular cyanobacterium *Synechocystis* sp. strain PCC 6803 and the chemoheterotroph *E. coli* K12 were used to reproduce a laboratory-scale dual species SAB on limestone, as calcareous stone materials are typical CH surfaces with high bioreceptivity (Miller et al., 2012).

These two microorganisms have been previously retrieved on stone heritage (*inter alia* Ortega-Morales et al., 2000; Bellinzoni et al., 2003; Crispim et al., 2003; Bartolini et al., 2004; Ikner et al., 2007; Pereira de Oliveira et al., 2008; Macedo et al., 2009; Bastian et al., 2010), highlighting their occurrence in biofilm at the stone/air interface.

The selected model organisms are fast-growing microorganisms, enable the design of replicated experiments across a wide range of spatial and temporal scales, and to better explore biofilm responses to environmental changes in a reasonable timescale. Furthermore, they have a number of other important advantages, namely a well-developed literature base, being genetically tractable and amenable to molecular technique such as mutagenesis and “omics” based approaches, existence of *in silico* metabolic models, as well as relevance to bioremediation and biomineralization (Ikeuchi and Tabata, 2001; Hayashi et al., 2006; Yoshikawa et al., 2011; Han et al., 2013).

Although *Synechocystis* and *E. coli* are components of the biofilm community inhabiting stone heritage, they direct interactions have never been explored. In the proposed lab scale system we forced the two microorganisms to interact with each other in an oligotrophic environment, providing the platform to address many important questions about the mechanisms of species interactions on stone.

The DFR has been previously successfully applied to modeling biofilms in industrial piping systems, catheters, wounds, lungs, and oral cavity environments (*inter alia* Goeres et al., 2009; Brindle et al., 2011; Woods et al., 2012; Tremblay et al., 2013). In this work, for the first time, the DFR was used to reproduce at laboratory scale biofilms at the stone-air interface. The DFR can be modeled as a plug flow reactor in which cell density and nutrient concentration change along the length of the coupon (Goeres et al., 2009). The wall of stone heritage exhibits the properties of an open plug flow reactor. SABs on outdoor stone monuments show a typical colonization pattern, following the water flow downward. Biofilms inhabiting stone monuments experience low fluid velocity over the surface, and the biomass is continuously exposed to the air. In the same way, biofilms in the DFR are under low-shear/laminar flow as the medium drips onto a surface set at a 10° angle, and high gas transfer environment as the biofilm is continuously exposed to the air in the head space (Woods et al., 2012). The system used in this work can be easily adapted to mimic a variety of environmental conditions. By simply modifying the composition and the flow rate of the influent, it is possible to simulate different environmental scenarios such as acid rain, drought, increase in salinity, increase in rainfall events etc. The gas in the headspace can be varied, and the reactor can be accommodated inside an environmental chamber to control intensity of the incident radiation and the experimental temperature.

**FIGURE 1:**
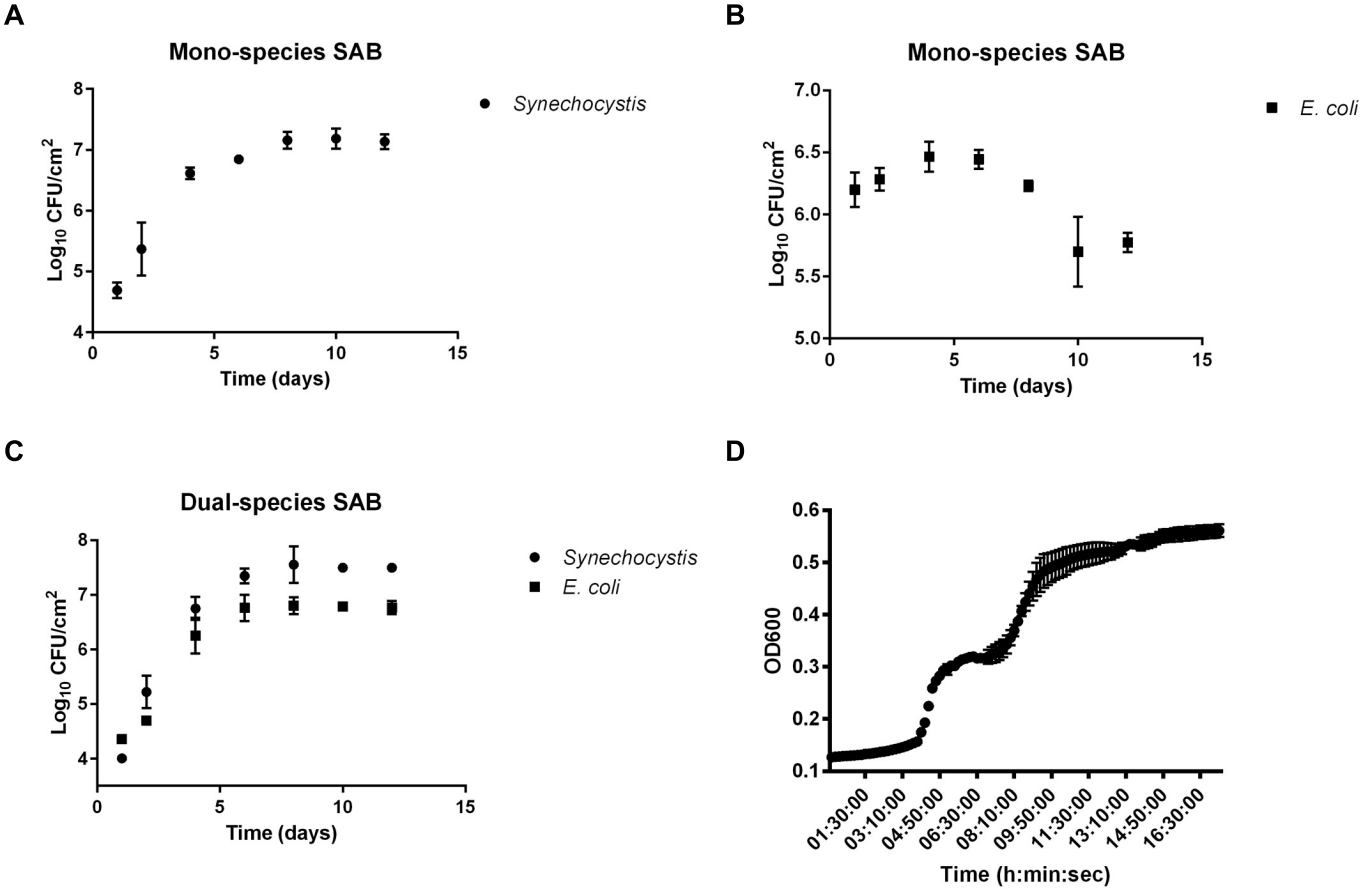
**Biofilm growth curves.** Biofilm growth of *Synechocystis* and *Escherichia coli* in mono (**A,B**, respectively) and co-culture **(C)**. Data points are average of triplicate experiments. Error bars represent standard deviation of experiments. Panel **(D)** shows the growth of *E. coli* on cyanobacterial EPS. Data points are average of triplicate experiments. Error bars represent standard deviation of experiments.

As a first step, we sought to examine the biofilm growth of *Synechocystis* and *E. coli* alone and in combination. The results demonstrated the ability to grow dual-species SABs as well as mono-species photoautotrophic ones, but not mono-species chemoheterotrophs, which lack carbon-fixing ability (**Figures 1A–C**). After 24 h of batch condition, the attached cyanobacterial cells in the mono-species SAB averaged 6.2 ± 0.14 Log_10_ CFU/cm^2^ compared to just 4.01 ± 0.06 log_10_ CFU/cm^2^ in the dual-species SAB. The decreased attachment success of both *E. coli* and *Synechocystis* in the dual-species biofilm can be explained taking into account the competitive attachment phenomenon. Attachment by different bacterial species in mixed culture suspensions might be expected to be competitive, thus resulting in reduced attachment of the component species (Lappin-Scott and Costerton, 1995).

Despite the initial difference in the number of attached cells, plates count showed that mono-species *Synechocystis* SAB formed less rapidly (1.31 ± 0.19 log_10_ CFU/day) than the dual-species SAB (1.66 ± 0.084 log_10_ CFU/day). This disparity was even more strikingly evident at the 8th day, where the cell numbers in the dual-species SABs were higher than in mono-species SABs, both for *Synechocystis* and *E. coli*. At the 10th day, the SABs appeared similar to the 8th day, suggesting that a ‘steady state’ had been reached.

Observation in field demonstrates that SABs on stone heritage are dominated by associations of phototrophic and heterotrophic microorganisms (Albertano and Urzì, 1999; Cappitelli et al., 2012; Polo et al., 2012). The accumulation of photosynthetic biomass provides an excellent organic nutrient base for subsequent heterotrophic microbiota and their biodeterioration activities (Crispim and Gaylarde, 2005). Phototrophs facilitate the establishment of a complex SAB community by excreting carbohydrates and growth factors (Crispim and Gaylarde, 2005; Dakal and Cameotra, 2012). In subaerial environments, an important class of interactions is based on cross-feeding and metabolic exchange, whereby photosynthetically fixed dissolved organic carbon sustains the growth of heterotrophic bacteria (Cole et al., 2014; Valverde et al., 2015). The heterotrophs, in turn, can promote cyanobacterial growth by providing key metabolites and scavenging waste products (Morris et al., 2008; Hayashi et al., 2011; Beliaev et al., 2014). In this way, the metabolic capacity of the consortium expands and improves resource utilization efficiency in comparison to its individual members (Cole et al., 2014).

In oligotrophic conditions, cyanobacterial extracellular polymeric substances (EPS) represent a notable source of organic carbon available for cross-feeding processes (Rossi and De Philippis, 2015). In this work, we tested whether *E. coli* could use the biofilm EPS produced by *Synechocystis* as a carbon and energy source, laying the foundation for one of the possible mechanisms behind the cross-feeding processes. The EPS extracted from the mono-species cyanobacterial SAB was used as a culture medium to sustain the planktonic growth of *E. coli*. The results showed that the EPS of *Synechocystis* presented a polysaccharide content of 21.68 μg/mg_dry biomass_ that can sustain the planktonic growth of *E. coli* (**Figure 1D**). Interestingly, the heterotrophic bacteria exhibited a diauxic growth, in which the preferential carbon source is consumed in the first growth phase, whereas the less favorable secondary carbon source supports growth during the second growth phase.

### Microscopy

**FIGURE 2:**
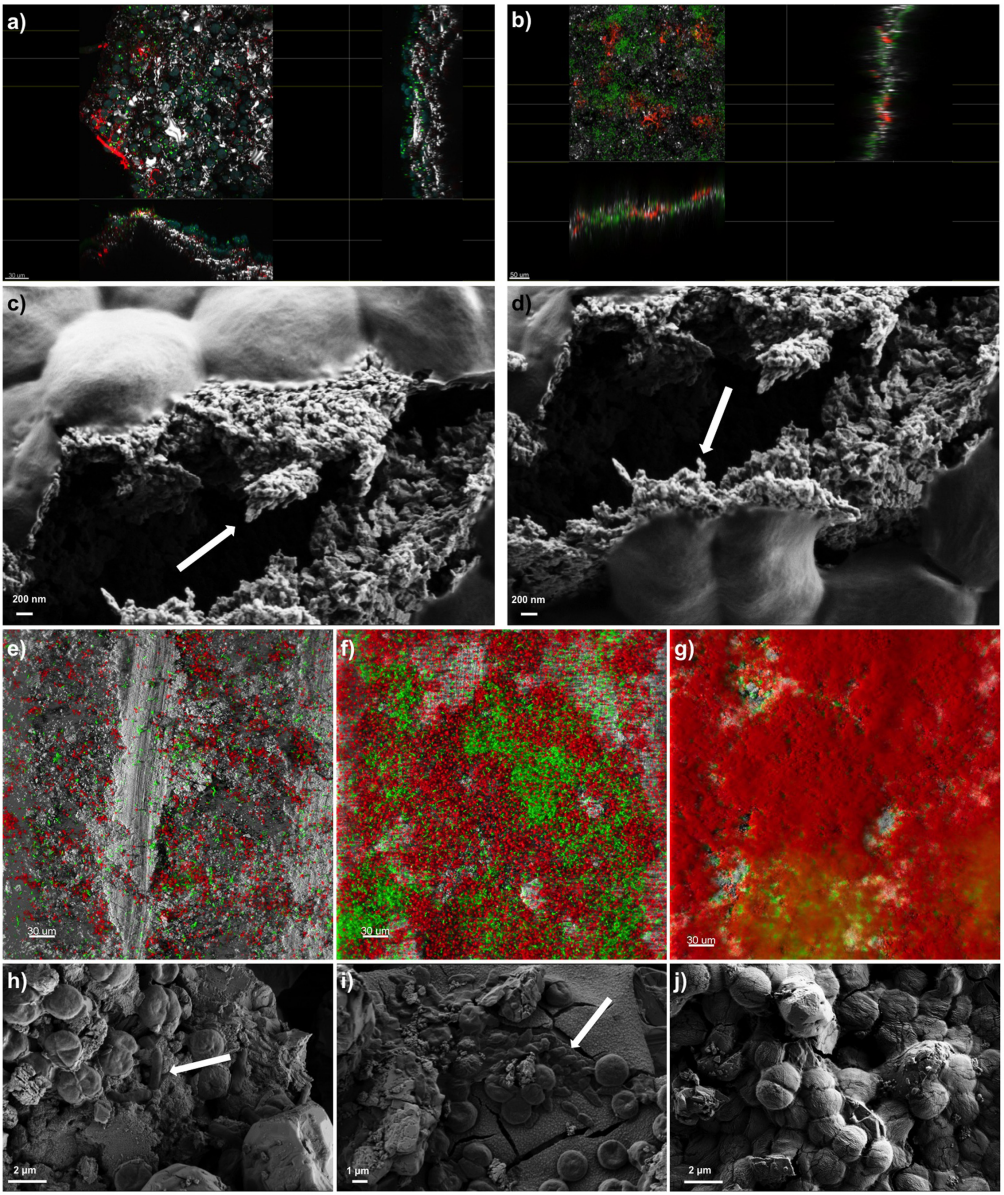
**Biofilm imaging by Confocal Laser Scanning Microscopy (CLSM) and Field Emission Scanning Electron Microscopy (FE-SEM).** Panel **(a)** shows the extended view (z-y and z-x planes) of a SAB colonizing the marble surface of the Lincoln Memorial (Washington, DC, USA). Scale bars represent 30 μm. Color key: heterotrophs, green; Phototrophs, blue; EPS-labeled ConA, red; reflection from inorganic materials, gray. Panel **(b)** represents the extended view (z-y and z-x planes) of a mature dual-species SAB. Scale bars represent 50 μm. Color key: *Synechoscystis* cells, green; EPS-labeled ConA, red; reflection from inorganic materials, gray. Panels **(c,d)** show the EPS of a mature dual-species SAB. Arrows indicate the fibriform extracellular matrix-like structures. Panels **(e–g)** display the development of a dual-species SAB over time monitored by CLSM. Scale bars represent 30 μm. Color key: *E. coli* cells, green; *Synechocystis* cells, red; reflection from inorganic materials, gray. Panels **(h–j)** show the FE-SEM micrographs of cells arrangement during the development of the dual-species SAB over time. Arrows indicate the rod-shaped *E. coli* cells. The images reported are representative of different images taken from independent experiments.

The presence of a polysaccharide-rich matrix was further confirmed by images captured from CLSM combined with lectin staining. The fluorescently labeled Concanavalin A (ConA), has been widely used to characterize glycoproteins and other sugar-containing entities on the surface of various cells as well as in the biofilm EPS (Roldán and Hernández-Mariné, 2009; Villa et al., 2012). Microscopic observations of SABs inhabiting real stone monuments revealed the presence of ConA-labeled EPS in contact with the lithic substratum, and the biomass lying on the top of the visualized matrix (**Figure 2a**). In the same way, ConA signal from the lab-scale SAB mainly accumulated on the bottom of the biofilm, showing the intimate connection with the stone as the signal spread between the mineral grains filling depressions, fissures and intergranular spaces (**Figure 2b**). Field Emission Scanning Electron Microscope (FE SEM) images further confirmed the presence of amorphous materials -consistent with an EPS layer in contact with the stone (**Figures 2c,d**).

It has been well established that cyanobacteria produce EPS mainly of polysaccharidic nature (De Philippis et al., 2001; Pereira et al., 2011). The monosaccharides most frequently found in cyanobacterial EPS are fucose, rhamnose, arabinose, galactose, glucose, mannose, xylose, galacturonic acid and glucuronic acid (Rossi and De Philippis, 2015). The complex carbohydrates act as barriers against different type of stress and play a role in intra- as well as inter-species interactions (Kehr and Dittmann, 2015). It is a fact that cyanobacteria isolated from stone monuments excrete large amounts of EPS as an adaptation to drought (Macedo et al., 2009; Rossi et al., 2012; Vázquez-Martínez et al., 2014). In addition, the presence of a large number of different monosaccharides in the cyanobacterial EPS represents a considerable trophic resource for the epilithic community when polymers are degraded (Flemming and Wingender, 2010; Rossi et al., 2012). For instance, a study of EPS turnover in modern stromatolites reported that 3–4% of the total carbon fixed through photosynthesis was incorporated in newly produced EPS, and that 40–60% of this new EPS was degraded to CO_2_ by heterotrophic bacteria within 24 h (Decho et al., 2005).

Confocal Laser Scanning Microscopy and image analysis of biofilms formed by the fluorescent protein-tagged *E. coli* and autofluorescent *Synechocystis* were used as non-invasive tools to investigate the development and the structure of dual-species SABs. Different projections were generated by Imaris software package for 3D reconstruction of cell aggregates (**Figures 2e–g**). The investigation revealed discrete colonies of *Synechocystis* surrounded by loose assemblages of *E. coli* at day 1 (**Figure 2e**). At day 6 the association changed, showing aggregation of *E. coli* cells in small colonies surrounded by cyanobacterial cells (**Figure 2f**). Within a few more days, *E. coli* colonies had been overgrown by a mantle of *Synechocystis* (**Figure 2g**). FE SEM analyses further corroborated the colonization pattern previously described (**Figures 2h–j**). The dual-species SAB appears patchy or network like, following the topography of the surface.

Ramirez et al. (2010) reported stratifications of SABs developed on the stone, stucco and mortar of El Palacio wall (Mexico). In particular they observed that the lower portion of the SABs contained coccoid and colonial cyanobacteria as well as other bacteria, whereas the upper portions encompassed mainly filamentous cyanobacteria, coccoid cyanobacteria and green algae. Thus, the phototrophic component of the SABs resides in contact with the air, covering the heterotrophic biomass. This stratification could be rationalized by not only considering the light and CO_2_ requirements of cyanobacteria, but also their capacity to survive harsh environmental conditions (e.g., desiccation, UV radiation) while offering protection to sensitive microorganisms. In aerial microbial mats, UV tolerant species like cyanobacteria normally occupy the mat surface, giving protection to the more sensitive species below (Norris et al., 2002).

On lithic surfaces, phototroph-heterotroph associations produce different colonization patterns and appear as a patchy distribution of cells that accumulate in fissures, cracks, or subsurface and deep layers, depending on the porosity and state of conservation of the material, as well as on the ecological requirements of individual species (Urzì and Albertano, 2001; Gorbushina, 2007; Gorbushina and Broughton, 2009). Furthermore patchy SABs serve as condensation and water collecting points, where liquid water and water vapor are not only quickly absorbed by the biofilm, but also retained for longer periods than on the neighboring rock surface (Gorbushina, 2007).

Cryosections of the dual-species SABs combined with microscopy revealed a biofilm thickness of 32.27 ± 9.21 μm, in line with in field observations of SABs on stone monuments. Ramirez et al. (2010) investigated the phototrophic SABs inhabiting a Mayan monument in Palenque, Mexico. The microscopic investigations revealed a biofilm thickness ranging from 21.8 to 64.5 μm. The SABs inhabiting three Spanish caves had a thickness ranging from 6.45 to 25.47 μm, and the biofilm thickness decreased with decreasing light (Roldán and Hernández-Mariné, 2009).

### Microelectrode Measurements

**FIGURE 3:**
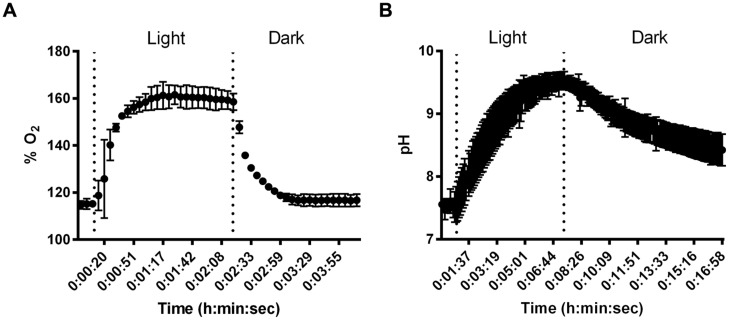
**Results of changes in O_2_ % saturation **(A)** and pH **(B)** in dual-species SABs during shifts between light and dark conditions.** Data points are average of triplicate experiments. Error bars represent standard deviation of triplicate experiments.

Despite small thickness and low biomass, SABs on stone monuments show high physiological capabilities. Microelectrode measurements were carried out to give experimental evidence of the effect of cyanobacterial photosynthesis on oxygen production and pH variation in dual-species SABs. The dissolved O_2_ (DO) measured as % saturated DO at the biofilm surface during a light/dark cycle is shown in **Figure 3A**. The increase of oxygen concentration occurred immediately after the start of the illumination. The production of oxygen increased over time, reaching a plateau after few minutes of light exposure (**Figure 3A**). The results of pH measurements for dual-species SABs showed that starting from near neutral values (pH 7.4) the H^+^ concentration increased about 2 pH units (pH 9.6) in the transition from dark to light (**Figure 3B**). The microelectrode measurement was integrated over a depth of approximately 20–25 μm, thus the measured DO and pH are representative of the conditions throughout most of the biofilm depth. In cyanobacterial communities, intensive photosynthesis results in a sharp increase of pH due to the consumption of protons due to equilibration between CO_3_^-2^ and HCO_3_^-^ (Albertano et al., 2000; Dhami et al., 2014).

Measurements of pH showed that, starting with values slightly below neutral, the pH in cyanobacterial biofilms increased by 0.24–0.77 units in the transition from dark to 1000 μmol photon m^-2^ s^-1^ irradiance (Albertano et al., 2000).

The variation from neutral pH values during the dark period to alkaline pH during illumination occurred to a sufficient extent to possibly induce precipitation of mineral compounds, especially in calcareous substrata (Albertano et al., 2000). The microelectrode measurements demonstrated that photosynthetic/respiration activity of the dual-species SAB induced variation in the chemical parameters that characterize the microhabitats of lithic sites.

### Desiccation Recovery

**FIGURE 4:**
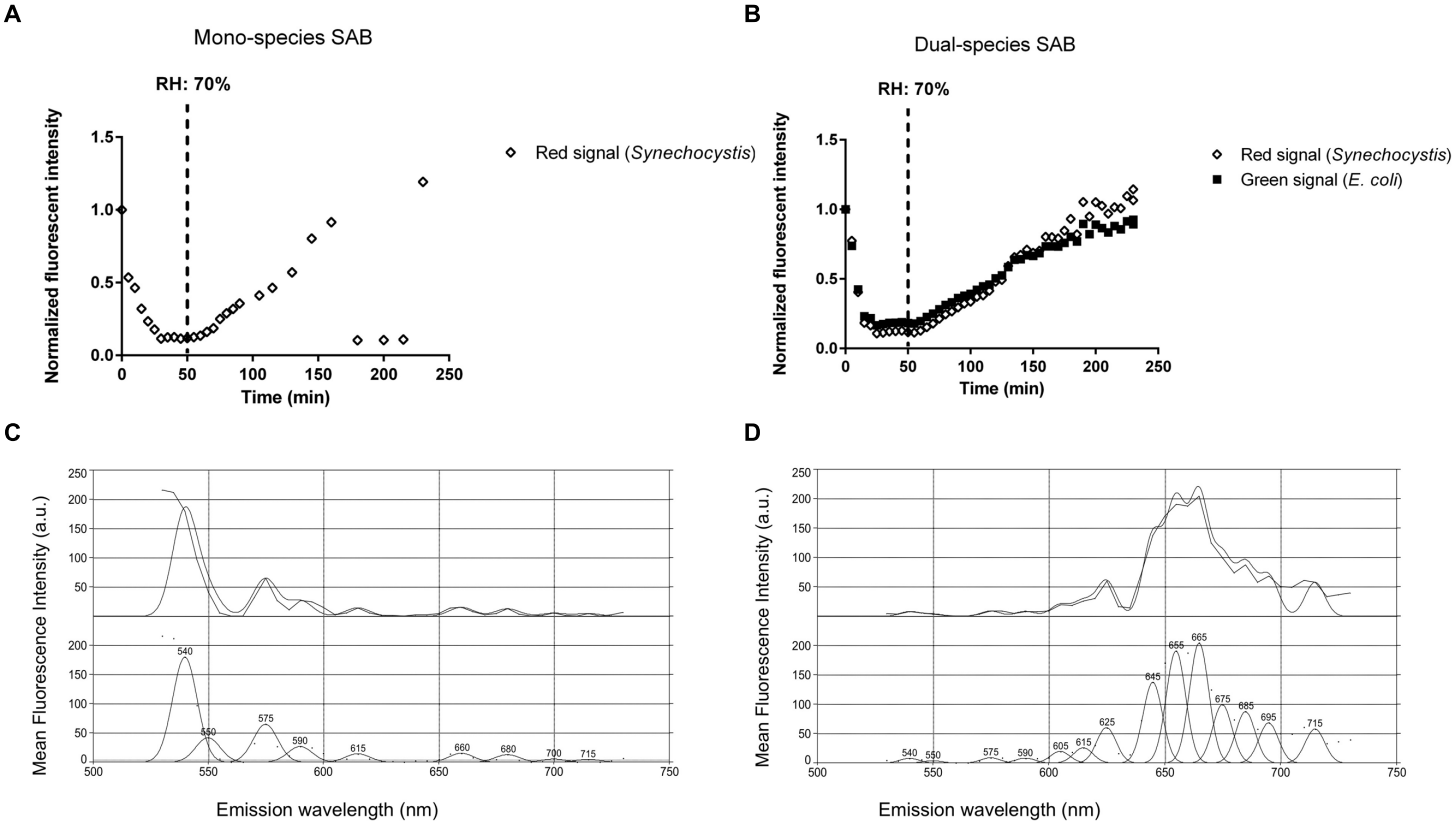
**Biofilm recovery after desiccation.** Recovery of fluorescence of a mono **(A)** and dual-species SAB during rewetting **(B)**. Emission spectra recorded in cyanobacterial cells before **(C)** and after **(D)** rewetting. Spectral profiles were analyzed by PICKFIT deconvolution software to show individual phycobiliproteins: Phycoerythrin, 575 nm; Phycocyanin, 645 nm; Allophycocyanin, 665 nm; Chlorophyll *a*, 685 nm (Wolf and Schübler, 2005).

Periods of desiccation and rewetting are regular, yet stressful events encountered by SABs on stone monuments. To examine the recovery of SABs following dehydration, mono- and dual-species SABs were allowed to desiccate for 1 h under a stream of sterile air (25% RH), followed by exposure to atmospheric moisture in form of humid air (90% RH). Live cell imaging showed that during rewetting the recovery of both GFP (*E. coli*) and red chlorophyll fluorescence (*Synechocystis*) was simultaneous and it occurred within minutes when the RH rose above 70% (**Figures 4A,B** and **Video 1** in Supplementary Materials). In photosynthetic microorganisms, autofluorescence of photosynthetic pigment is considered an indicator of cyanobacterial cell viability showing the integrity of the photosynthetic apparatus (Billi et al., 2011; Roldán et al., 2014). GFP has already been applied as an indicator for cellular viability in both bacteria and yeasts (Lehtinen et al., 2003; Mohan et al., 2013; Hoogenkamp et al., 2015). Furthermore, the fluorescence emission spectra recorded in cyanobacterial cells after desiccation showed a weak fluorescence within the green range indicating degraded photosynthetic pigments (**Figure 4C**, Roldán et al., 2014). After rewetting, cyanobacterial cells exhibited a spectral profile corresponding to the emission peaks of phycobiliproteins and chlorophyll *a*, corroborating the recovery of the photosynthetic apparatus from desiccation (**Figure 4D**, Wolf and Schübler, 2005; Roldán et al., 2014).

Desiccation tolerance is well documented in the literature for cyanobacteria (Gorbushina and Broughton, 2009; Keshari and Adhikary, 2013). *Nostoc* and *Chroococcidiopsis*, two of the main genera retrieved on stone monuments, were found to survive repeated cycles of desiccation and rehydration (Crispim et al., 2003; Tamaru and Takani, 2005), preserve the structural integrity of their cell structures after many years of storage in a dry state (Billi et al., 2013), and resume respiration and photosynthesis within minutes after rewetting (Wynn-Williams, 2000; Abed et al., 2014). SABs are highly water absorbent and are rapidly hydrated by atmospheric moisture available in form of rain, dew, fog, and humidity (Gorbushina, 2007). Recently, Davila et al. (2013) observed that the photosynthetic systems of endolithic cyanobacteria found in halite nodules in the hyperarid core of the Atacama Desert were inactive below a RH of 60%. However, when the RH rose above 70% and the salt became wet by way of deliquescence, fluorescence appeared within minutes, suggesting that the cyanobacteria are optimally adapted to ‘power up’ and ‘power down’ rapidly to take advantage of available moisture (Davila et al., 2013).

### Biocide Tolerance

Biofilm resistance to biocides is becoming a global issue with an impact on many fields, including CH. Biofilms are commonly viewed as being resistant to killing by a broad range of antimicrobial agents. In these experiments we compared the susceptibility of planktonic and SAB cells to quaternary ammonium salt-based disinfection treatment.

**FIGURE 5:**
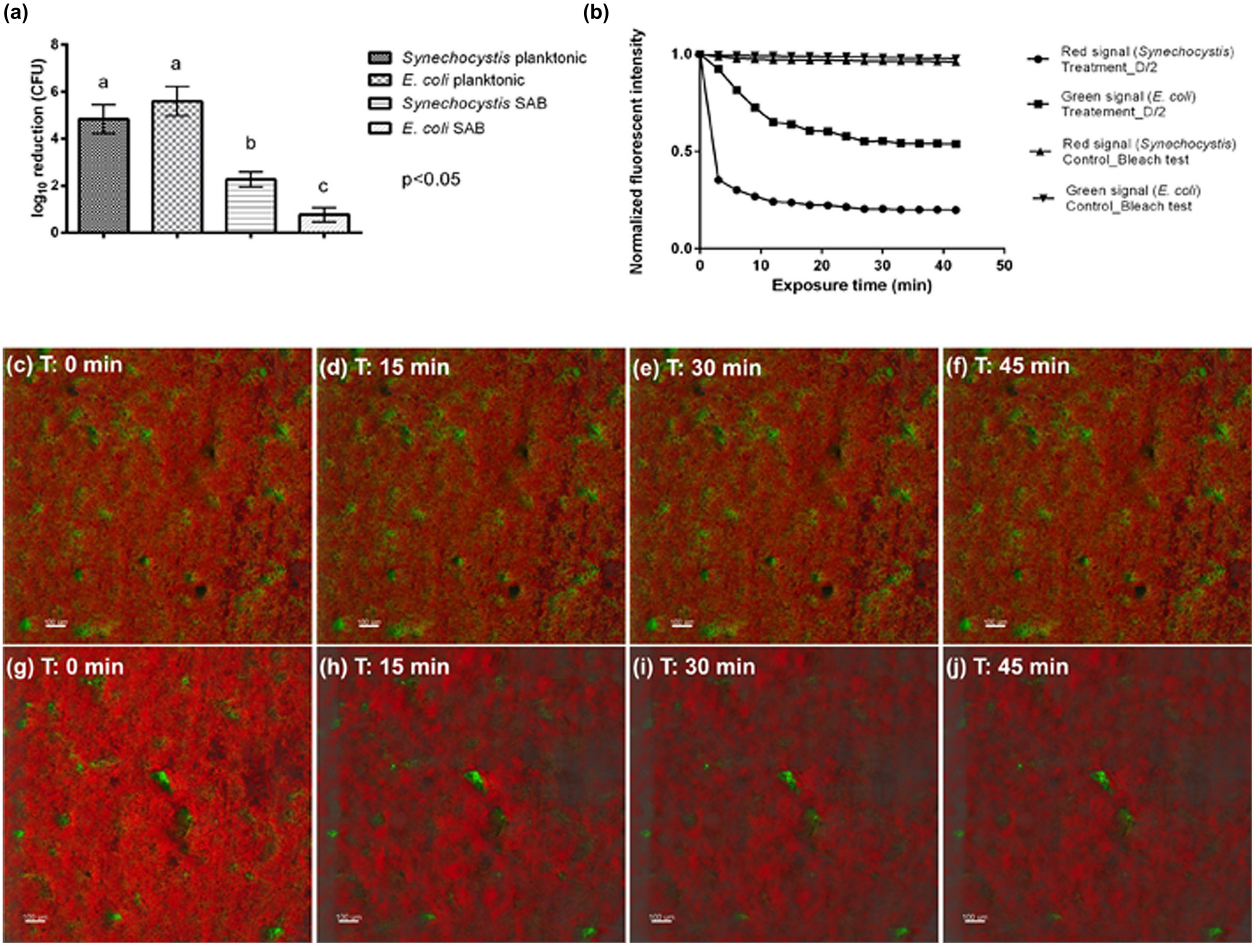
**Antimicrobial effectiveness of D/2 solution.** **(a)** Susceptibility of *Synechocystis* and *E. coli* in planktonic cultures and SABs observed as log_10_ reduction in the number of CFU after exposure to the antimicrobial agent. Data represent the mean ± standard deviation of three independent measurements. The letters above the histogram represent the results of Tukey’s *post hoc* comparisons of group means. According to *post hoc* analysis (*p* < 0.05), means sharing the same letter are not significantly different from each other. **(b)** Quantification of biofilm fluorescence intensity lost during exposure to the biocide. **(c–f)** Real-time loss in cell viability over time in absence of a biocide treatment (control sample). **(g–j)** Real-time loss in cell viability over time in presence of the biocide treatment (treated sample). Color key: *E. coli* cells, green; *Synechocystis* cells, red.

The log inactivation results based on viable plate counts are summarized in **Figure 5a**. As expected, the dual-species SAB showed more tolerance to antimicrobials than do planktonic cells. These modest log reductions, after 45 min of antimicrobial exposure, suggest that the dual-species SAB captures the antimicrobial tolerance that is a hallmark of the biofilm mode of growth. The overall loss of fluorescence in cell clusters after 45 min of exposure to the treatment agent was also recorded (**Figures 5g–j** and **Video 2** in Supplementary Materials). The extent of fluorescence loss was 80 and 46% for *Synechocystis* and *E. coli*, respectively (**Figure 5b**). The control experiment showed that both the green and red fluorescence dropped less than 5% in absence of the biocide treatment (**Figures 5c–f**). Thus, the fluorescence loss observed in presence of D/2 can be ascribed to the treatment.

Interestingly, both the plate count and the time-lapse CLSM analyses showed that the biocide treatment affected mainly the phototrophic component of the dual-species biofilm, suggesting protection of the heterotrophic population is provided by the cyanobacteria. It has been well recognized that different factors affect biofilm tolerance to antimicrobial agents, including the effects of the limited penetration of antimicrobial agents, changes in the bacterial phenotype of biofilm cells, as well as biofilm cells in persister states (Hall-Stoodley et al., 2004). In addition, after antimicrobial treatment the biofilm community may be turned into one that is resistant to those particular biocides, exerting even more harmful effects on the object of art. For instance, in the Lascaux Caves, the long series of biocide treatments triggered the development of resistant strains with biodeteriogenic properties (Martin-Sanchez et al., 2012).

## Conclusion

The primary accomplishment of the work described herein is the development of a methodology to obtain a new *in vitro* model of a fast-growing, phototroph-heterotroph mixed species biofilm at the stone/air interface.

The experiments reported underscore the ability of the dual-species SAB model to capture functional traits characteristic of biofilms inhabiting lithic substrata such as: (i) microcolonies of aggregated bacteria; (ii) a network like structure following surface topography; (iii) autotroph-heterotroph interactions; (iv) the ability to change the chemical parameters that characterize microhabitats; (v) survival in harsh environment; and (vi) biocide tolerance.

The inherent features of this biofilm are typical for any natural biofilm. However, to the best of our knowledge, this is the first time that all these properties have been proved in a lab-scale system mimicking phototroph-heterotroph mixed species biofilm at the stone/air interface.

Distinguishing characteristics that make the developed SAB system widely applicable as compared to other systems available for research purposes include the following: (i) a commercially available bioreactor, (ii) microorganisms genetically tractable and amenable to mutagenesis and “omics” based approaches, (iii) fast-growing biofilm, and (iv) adaptability of the system to a variety of different environmental conditions.

We should emphasize that bare rock surfaces are habitats for highly adapted, strongly melanized, slow growing Ascomycetes that are highly relevant to CH studies as well as other groups such as actinobacteria and nitrifying bacteria. Despite the precise choice to use *Synechocystis* and *E. coli* as representatives of the phototrophic and heterotrophic biofilm community, the present system can be adapted to host different microorganisms, including the most ecologically relevant melanized fungi, actinobacteria and nitrifying bacteria.

We would like to point out that this laboratory model system is not intended to be a miniaturized version of field systems. Rather, the purpose of the laboratory model systems is to simplify nature so that it can be more easily understood. If we cannot accurately predict the behavior of a simplified laboratory system, it is unlikely we can understand enough to make predictions of field systems (Jessup et al., 2004).

With its advantages in control, replication, range of different experimental scenarios and matches with the real ecosystem, the developed dual-species SAB model system is a particularly powerful tool to advance our mechanistic understanding of the spatial-temporal patterns of interactions between biofilm, stone and the atmosphere. Understanding these interactions is crucial to addressing ecological and biogeochemical questions, as well as developing tools to predict and model biodeterioration/bioprotection processes on lithic surfaces.

In addition, the present lab-scale system has the potential to mimic SAB inhabiting rocks in hyperarid zones or biological soil crusts. This lab-scale model system provides an elegant ecological framework for deciphering interspecies interactions, principles of microbial community assembly, biofilm biology, biogeochemical processes, and feedback responses to climate change.

## Conflict of Interest Statement

The authors declare that the research was conducted in the absence of any commercial or financial relationships that could be construed as a potential conflict of interest.

## Supplementary Material

**VIDEO 1 | Time lapse Confocal Laser Scanning Microscopy (CLSM) of biofilm recovery after desiccation.** The method is based on the monitoring of fluorescence recovery of the dual-species SAB during rewetting. Live cell imaging showed that during rewetting the recovery of both GFP (*Escherichia coli*) and red chlorophyll fluorescence (*Synechocystis*) was simultaneous and it occurred within minutes when the relative humidity rose above 70% (50 min).

**VIDEO 2 | Time lapse CLSM of D/2 action was performed in the dual-species SAB.** This technique permits the direct visualization of cell inactivation patterns in biofilm structures during the biocide action. The method is based on the monitoring of fluorescence loss from GFP *E. coli* cells and autofluorescence loss from *Synechocystis* cells, used to monitor real-time loss in cell viability.
